# Efficient modeling of neural activity using coupled renewal processes

**DOI:** 10.1186/1471-2202-12-S1-P123

**Published:** 2011-07-18

**Authors:** Felipe Gerhard, Wulfram Gerstner

**Affiliations:** 1 Brain Mind Institute, Ecole Polytechnique Fédérale de Lausanne, 1015 Lausanne EPFL, Switzerland

## 

Generalized Linear Models (GLMs) are stochastic models that have been successfully used to model neural activity of single cells and populations. Typically, this requires the spike train to be binned into a binary time series that is modeled as a Bernoulli-type GLM. Unless a loss in temporal precision is acceptable, the bin size has to be chosen on the order of milliseconds. This renders an application to long-term recordings infeasible for computational reasons.

We propose a new, binning-free application of the GLM framework to model neural activity by noting that spike trains can equally be characterized by the sequence of continuous-valued inter-spike intervals (ISIs). It was shown that single-neuron spike trains can often be sufficiently modeled as a non-stationary renewal process. Here, we focus on the Gamma distribution for the inter-spike intervals as it allows the accommodation of a relative refractory period and includes the inhomogeneous Poisson process as a special case. We generalize the idea of a modulated Gamma process to the population level by including cross-couplings between the neurons in case of recordings from multiple neurons in parallel.

In a Gamma-GLM, each observed inter-spike interval is modeled as a sample from a Gamma distribution whose mean can vary with time, e.g. through modulation by an external stimulus. Traditionally, ISIs are assumed to be independent. However, this renewal assumption can be relaxed by conditioning on the durations of previous intervals. An inter-neuron coupling is introduced by additionally conditioning on the times of spikes of the other neurons. The Gamma distribution and other canonical distributions for inter-spike intervals are part of the exponential family so that efficient maximum likelihood solutions for all model parameters can be obtained using the framework of Generalized Linear Models. Considering the ratio between bin sizes for time-discrete models and the typical length of cortical inter-spike intervals, the computational demand can be reduced by several orders of magnitude. Goodness-of-fit is assessed by adapting standard tests for point process models and evaluating spike prediction performance based on cross-validation.

We demonstrate the versatility of the coupled Gamma model for estimating functional connectivity from multi-electrode recordings. With a data set obtained from multi-electrode recordings of the visual system of the awake monkey, we show that the test power of the Gamma-GLM for detecting functional connectivity links is comparable to the performance of a classical discrete-time GLM albeit requiring much less computational time and memory resources. Using the two models on the same data set, we are able to estimate the unknown, underlying connection density of the network. Finally, we discuss general limitations of coupled Gamma-GLMs on the type of interactions that can be faithfully represented and discuss similarities to other binning-free models, in particular Cox’s proportional hazards model.

